# Supplementary material for: Evidences of histologic thrombotic microangiopathy and the impact in renal outcomes of patients with IgA nephropathy
Source: PLoS One. 2020 Nov 4;15(11):e0233199. doi: 10.1371/journal.pone.0233199 (PMC7641451; doi:10.1371/journal.pone.0233199)
Supplement: Attachment — Submitted filename: Neves et al_LettertoReviewers_2020.docx [file pone.0233199.s002.docx]

|  | UNIVERSIDADE DE SÃO PAULO  FACULDADE DE MEDICINA  Av. Dr. Arnaldo, 455 - 4º andar - Sala 4304 - CEP 01246-903  São Paulo - SP - Brasil  Phone: 11-3061-8399 - Fax: 11-3061-8361 |
| --- | --- |

September 16^th^, 2020

To Dr. Joerg Heber

Editor-in-Chief of PLos One

Dear Editor,

Please find attached the revised version of the manuscript ***“The impact of histologic evidence of thrombotic microangiopathy in the renal outcomes of patients with IgA nephropathy***”, by Neves et al, which is being submitted for the “Original Research Article” section.

We thank you for the follow-up and the reviewers for the helpful comments and suggestions, which certainly improved our manuscript quality.

We believe that we have appropriately addressed all points raised by the reviewers and that the manuscript is now suitable for publication. The specific responses to the editors and reviewers are outlined below.

**REVIEWER 1 REPORT:**

**Thank you for addressing reviewers comments. It is an interesting topic, and I think this revised version has improved manuscript content.**

**Response:** Thank you for your kind comment. Improve the quality of our manuscript was just possible once you have been contributed with your valuable suggestions.

**REVIEWER 3 REPORT:**

**1. Almost all comments have been addressed appropriately. However, I would recommend to exclude patients with no mesangial hypercellularity (M0) and no other morphologic features included in the MEST-C classification. This is particular the case if electron dense deposits were not found/electron microscopy was not performed.**

**Response:** Thank you for your relevant comment We do understand your concern including patients with MEST-C score zero (M0/E0/S0/T0/C0) in our protocol particularly lacking electronic microscopy analysis. Those patients could be misdiagnosed as IgAN instead of C3 Glomerulopathy (C3GP) . It is well defined that light microscopy (LM) and immunofluorescence analysis (IF) are sufficient for IgAN diagnosis while MEST-C score adds morphological parameters to clinical prognosis and not to diagnosis.

MEST-C score shows high variation among studies. In Valiga study (N=647) and North American Validation study (N=87) M0 is described in 73% to 10% of the patients, respectively, E0 from 88% to 69%, S0 from 25% to 35%, T0 from 78% to 80%. C parameter was added to MEST score recently and C0 is described in 80 to 90 % of the patients.

In our 118 IgAN patients, MEST-C parameters distribution M0 20.4% , E0 64.4% , S0 29.7% , T0 61.7% , C0 71.2 % was not different from world’s report while 8 patients showed MEST-C score zero in each parameter concurrently. Three out of those 8 patients did not show any C3 deposition making impossible the hypothetical diagnosis of C3GP that is defined as “C3 dominant and at least two orders of intensity stronger than any combination of IgG, IgM, IgA and C1q “ ( Jean Hou et al).

So far, 5 patients with C3 deposition and MEST-C score concurrently zero could be on hazard of a misdiagnosis of IgAN instead of C3GP (see table below describing each patient)

Going deeply on G3GP clinical features described on literature we can emphasize some characteristics :

- C3GP is an uncommon glomerulopathy when compared to IgAN.
- C3GP more frequent light microscopy pattern is Membranoproliferative (MPGN ) from 73% to 78 % (Caravaca-Fontan et al, and Avasare et al) while IgAN is mostly Mesangioproliferative, and in some patients depicts a FSGS pattern. No IgAN patient from our protocol discloses MPGN pattern.
- Considering IF findings in C3GP Avasare et al showed exclusive C3 deposition in 33%, C3+IgM in 17% and do not mention important IgA deposition while in IgAN that deposition is mandatory as disclosed in all our patients.
- Clinical evolution and prognosis are very different between IgAN and C3GP. Our questioned 5 patients showed on last follow-up visit , around 4 years, almost normal kidney function as expected in many non-progressive IgAN patients much different from C3GP patients reaching aroud 50% of kidney failure on the same time

**REFERENCES**

1. Jean Hou et al. Toward a working definition of C3 glomerulopathy by immunofluorescence. KI 85: 450-456, 2014
2. Caravaca-Fontan et al Mycophenolate Mofetil in C3 glomerulopathy and pathogenic drivers of the disease CJASN 15: 1287-1298, 2020
3. Avasare et al Mycophenolate Mofetil in combination with steroids for treatment ok C3 glomerulopathy : a case series CJASN 13: 406-413, 2018

We hope having cleared your question. Nevertheless we would be glad processing EM on those specific 5 patients. Unfortunately we have no biopsy material left.

We are Very gratefull for your comments that highly enriched our paper.

**Table: Clinical profile, laboratory tests, renal biopsy findings and outcomes of patients with M0, E0, S0, T0, C0 and C3+**

| **Clinical and Laboratory data at renal biopsy** | | | | | | | **Renal Biopsy Findings** | | | | | | | | | | | **End of follow-up** | | |
| --- | --- | --- | --- | --- | --- | --- | --- | --- | --- | --- | --- | --- | --- | --- | --- | --- | --- | --- | --- | --- |
| **N** | **Gender** | **SCr (mg/dL)** | **CKD-EPI**  **(ml/min/1,73m^2^)** | **Proteinuria**  **(g/dia)** | **HMT** | **HTN** | **M** | **E** | **S** | **T** | **C** | **TMA** | **IgG** | **IgA** | **C3** | **IgM** | **C1q** | **SCr**  **(mg/dL)** | **CKD-EPI**  **(ml/min/1,73m^2^)** | **Dialysis** |
| 4 | Male | 0,92 | 100 | 0,82 | Yes | No | 0 | 0 | 0 | 0 | 0 | No | neg | + | + | + | neg | 0,72 | 99,93 | No |
| 8 | Female | 0,7 | 92 | 0,51 | Yes | No | 0 | 0 | 0 | 0 | 0 | No | neg | + | + | neg | neg | 0,62 | 102 | No |
| 10 | Male | 0,75 | 113,6 | 1,39 | No | Yes | 0 | 0 | 0 | 0 | 0 | No | neg | + | + | neg | neg | 0,92 | 94,71 | No |
| 12 | Female | 0,8 | 91 | 0,33 | Yes | No | 0 | 0 | 0 | 0 | 0 | No | neg | ++ | + | neg | neg | 0,6 | 95 | No |
| 19 | Female | 1,2 | 52 | 2,5 | Yes | No | 0 | 0 | 0 | 0 | 0 | No | neg | + | + | + | neg | 1,1 | 55 | No |

**HMT: hematúria; HTN: hypertension; Neg: negative; SCr: sérum creatinine; TMA: Thrombotic Microangiopathy**

**2. Also, I would suggest to change the results section page 9 line 218 into: In total, 72 kidney biopsies were available to stain for C4d, including 9 tissue sections from patients with TMA and 63 tissue sections from those without TMA. Glomerular deposits of C4d were more frequent in patients with TMA as compared to those without TMA (n/M=7/9 and n/N=26/63; P=0.04).**

**Response:** Thank you for your suggestion. We have replaced the sentence as suggested (Page 9, lines 216-220).

Sincerely,

Precil Diego Miranda de Menezes Neves, MD

Division of Nephrology

University of São Paulo School of Medicine

São Paulo - Brazil
